# Multilevel functional data analysis modeling of human glucose response to meal intake

**Published:** 2024-05-23

**Authors:** Marcos Matabuena, Joe Sartini

**Affiliations:** Universidad de Santiago de Compostela and Department of Biostatistics, Harvard University, Boston, MA 02115, USA; Department of Biostatistics, Johns Hopkins University, Francisco Gude, Universidad de Santiago de Compostela

**Keywords:** continuous glucose monitoring, glucose metabolism, functional data analysis, personalized nutrition, multilevel models

## Abstract

Glucose meal response information collected via Continuous Glucose Monitoring (CGM) is relevant to the assessment of individual metabolic status and the support of personalized diet prescriptions. However, the complexity of the data produced by CGM monitors pushes the limits of existing analytic methods. CGM data often exhibits substantial within-person variability and has a natural multilevel structure. This research is motivated by the analysis of CGM data from individuals without diabetes in the AEGIS study. The dataset includes detailed information on meal timing and nutrition for each individual over different days. The primary focus of this study is to examine CGM glucose responses following patients’ meals and explore the time-dependent associations with dietary and patient characteristics. Motivated by this problem, we propose a new analytical framework based on multilevel functional models, including a new functional mixed R-square coefficient. The use of these models illustrates 3 key points: (i) The importance of analyzing glucose responses across the entire functional domain when making diet recommendations; (ii) The differential metabolic responses between normoglycemic and prediabetic patients, particularly with regards to lipid intake; (iii) The importance of including random, person-level effects when modelling this scientific problem.

## 1 Introduction

Recent advances in wearable technology and smartphones have revolutionized the collection of real-time longitudinal physiological data Kellogg et al. (2018), Dunn et al. (2018), Johnson et al. (2023). For example, continuous glucose monitoring (CGM) devices Rodbard (2016), Ebekozien et al. (n.d.) are minimally invasive, provide real-time glycemic response data, and can be used for managing diabetes Battelino et al. (2019), Matabuena et al. (2021, 2022), Beck et al. (2018), Rodbard (2018), Cui et al. (n.d.), as well as for screening diabetes in normoglycemic individuals and those with prediabetes Ben-Yacov et al. (2023), Hall et al. (2018a), Matabuena et al. (2022). This paper focuses on studying postprandial CGM data in individuals without diabetes, a key functional biomarker for assessing individual glucose homeostasis and metabolic health Mozaffarian et al. (2008), Berry et al. (2020), Merino et al. (2022).

There are significant limitations in the existing nutritional literature which can potentially be overcome by leveraging digital health data using appropriate methodologies, providing evidence for individual dietary recommendations. Historically, much dietary research literature has focused on subjective and self-reported measurements of diet, which have significant and unavoidable biases Maynard et al. (2005). Moreover, little is currently understood about the impact of meal composition on the glycemic response curve, with previous analyses not incorporating this information Wang et al. (2023). Despite decades of research in nutrition epidemiology, actual recommendations are highly variable, contradictory, and often confusing for the general population Archer (2017), Feinman (2019).

CGM devices provide a novel opportunity to characterize how an individual’s glucose responds to particular diets, but the most popular current statistical methods for analyzing these device data are insufficient Holzer et al. (2022). There is growing interest and consensus in using CGM devices to assess and improve personalized nutrition intervention. However, commonly-used existing methodologies for analyzing CGM data Berry et al. (2020) can be suboptimal for several reasons. Firstly, they often overlook the dynamics of glucose fluctuations over time—a critical factor for comparing individual glucose signatures across different people. They often focus instead on specific temporal points, such as glucose values three hours post-meal, or aggregated summaries over observation periods. Secondly, statistical inference can be far from ideal because it fails to incorporate the multilevel structure of the data, where multiple observations per individual are recorded. Thirdly, the modelling of random effects at the individual level is essential to accommodate the heterogeneity in postprandial responses between individuals.

**Figure 1:**
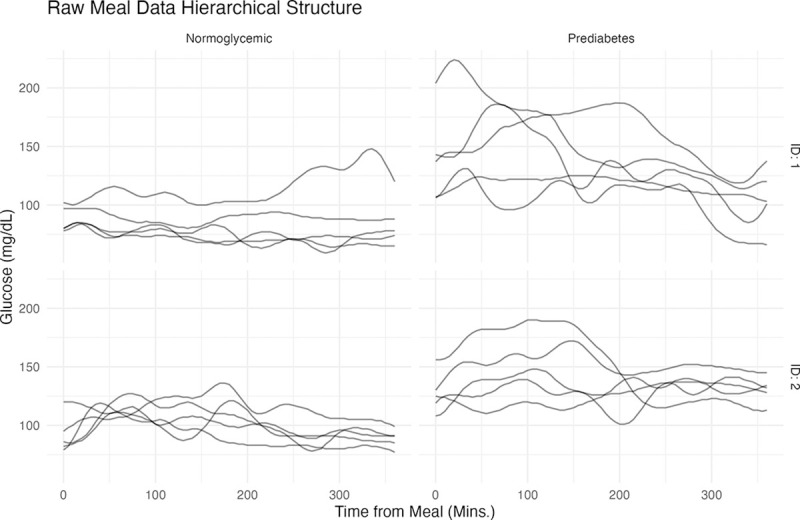
Analysis of post-meal functional data trajectories over several days in four distinct patients with different glycemic condition-normoglycaemic and prediabetes.

Functional analysis techniques Ramsay et al. (2005), Morris et al. (2001), Greven et al. (2010) can help alleviate these analytical limitations and improve our understanding of how glucose evolves over time as a mathematical function. To gain a deeper understanding of the advantages and the need of performing functional analysis, Figure 1 illustrates glycemic response curves over consecutive days post-dinner for four individuals—two normoglycemic and two with prediabetes. This figure highlights person-specific distribution patterns, with significant inter-individual and inter-day variability.

The final goal of this paper is to illustrate quantitative methods for addressing these scientific problems based on multilevel functional analysis techniques. We seek to investigate statistical associations between covariate predictors, including meal composition, and functional CGM postprandial responses. We also propose a new notion of R-square mixed functional coefficients to assess the time-dependent response function variability explained by the predictors and the individual-level random effects.

### 1.1 Summary of Contributions

The main methodological, modeling, and clinical findings of this scientific work are summarized as follows:

#### 1. Novel Statistical Modeling Strategy:

- We propose a novel statistical modeling approach for analyzing post-meal glycemic responses in patients. This method employs functional data analysis to expand the traditional use of classical mixed models for analyzing postprandial glycaemic data Berry et al. (2020).

#### 2. Methodological Contributions to Multilevel Functional Literature:

- Introduction of a new functional R-squared measure for both function-on-scalar regression and functional principal components models. This measure enhances our understanding of heterogeneity in the data by both calculating variance explained across the whole functional domain and facilitating its decomposition between the fixed and random effect components.
- Development of a two-step, semi-supervised algorithm to derive latent individual representations based on the multilevel functional mixed model. These representations can incorporate other patient characteristics and confounders which influence the multilevel functional process, e.g. diet, age, and body mass index.

#### 3. Clinical Findings from the AEGIS Study:

- Statistical association analyses between macro- and micro-nutrients and post-meal glucose response curves in individuals with normoglycemia and prediabetes. These analyses draw new conclusions concerning differential responses between the normoglycemia and prediabetes groups, underscoring the importance of considering the complete functional trajectory for nutritional recommendations.
- Application of the proposed functional mixed-model R-squared, offering new perspectives on model explanatory capacity over time and emphasizing the importance of incorporating random effects to capture individual glucose signatures.
- Utilization of functional data collected across different meals to identify unique signatures of glucose homeostasis, potentially useful in predicting long-term surrogate markers such as HOMA-IR.

### 1.2 Functional Modelling of CGM Data

The application of functional data analysis (FDA) to CGM data has emerged as a highly effective approach for various modeling tasks, gaining popularity in diabetes literature and related areas Matabuena et al. (2021, 2023, 2022), Cui et al. (n.d.), Gaynanova et al. (2020), Sergazinov et al. (n.d.). An important advancement in this area was introduced by Matabuena et al. with the concept of the glucodensity Matabuena et al. (2021), a novel representation of CGM distributions. This functional CGM profile, generalizing traditional CGM time-in-range metrics, provided novel insights in real-life monitoring scenarios Matabuena et al. (2023, 2022), Cui et al. (n.d.), Matabuena & Crainiceanu (2024).

In standardized environments, FDA techniques, when integrated with multilevel modeling, have proven highly effective Gaynanova et al. (2020), Sergazinov et al. (n.d.) in analyzing glucose patterns overnight in populations with diabetes. However, these types of multilevel models have yet to be applied in datasets of postprandial glucose responses among populations with normoglycemia and prediabetes. As highlighted in the introduction, this investigation into the structure of multilevel functional CGM responses to meals is becoming increasingly important in the field of personalized nutrition Berry et al. (2020). The reduction in the cost of CGM devices is expected to lead to their increased use among the general population as an accessible tool for assessing impacts of dietary choice.

## 2 The A Estrada Glycation and Inflammation Study (AEGIS)

The A Estrada Glycation and Inflammation Study (AEGIS) Gude et al. (2017), is a ten-year longitudinal study focused on changes in blood glucose and their connections to inflammation and obesity, factors critically linked to the potential development of comorbidities such as diabetes mellitus. Unlike traditional studies, AEGIS incorporates CGM for a subsample, providing detailed glucose profiles at various time points over a period of five years.

### 2.1 Study Design and Objectives

At the beginning of the study, a random sample from the general population of 1,516 individuals underwent extensive medical examinations to construct detailed individual clinical profiles. These included dietary habits, laboratory biomarkers, and responses to questionnaires assessing metabolic capacity, mental well-being, and lifestyles. CGM collection was completed for a subset of 581 participants, which included 516 individuals with normoglycemia and prediabetes, in a two-sample design. Of these individuals, 377 recorded at least one meal which the participant labelled as a ”dinner”; we focus on this subset.

The primary goal of our analysis was to examine the statistical association between dietary intake and associated postprandial glucose response, adjusting for patient characteristics, with a focus on evening meals identified as ”dinners”. To analyze glucose dynamics six hours post-meal, we employ a multilevel functional approach using multiple meals per individual over different days.

### 2.2 Data Description

The AEGIS trial (NCT01796184) involved a stratified random sample of individuals aged 18 and older, drawn from the Spanish National Health System Registry, providing a rich, decade-long longitudinal dataset.

#### 2.2.1 CGM Data Collection Protocol

Participants were fitted with Enlite^™^sensors and iPro^™^CGM devices, offering blinded interstitial glucose measurements every 5 minutes. On the seventh day, the sensor was removed, and data excluding the first day’s results were downloaded for analysis. Data from any day with more than 2 hours of data acquisition failure were discarded.

#### 2.2.2 Dietary Variables and Patient Information

Participants recorded their food and drink intake, which was validated by a research dietitian. To assess dietary intake, participants completed a 6-day food record that coincided with the CGM period. Detailed information was recorded regarding the types and amounts of foods and beverages consumed, including preparation methods, ingredients, sauces, and mealtimes. Data from 516 participants were considered for the final analysis given that 65 individuals were diagnosed with diabetes mellitus. Of these participants, only a subset of 377 recorded the meal of interest for these analyses.

### 2.3 Variables Description for Data Analysis

**Figure 2:**
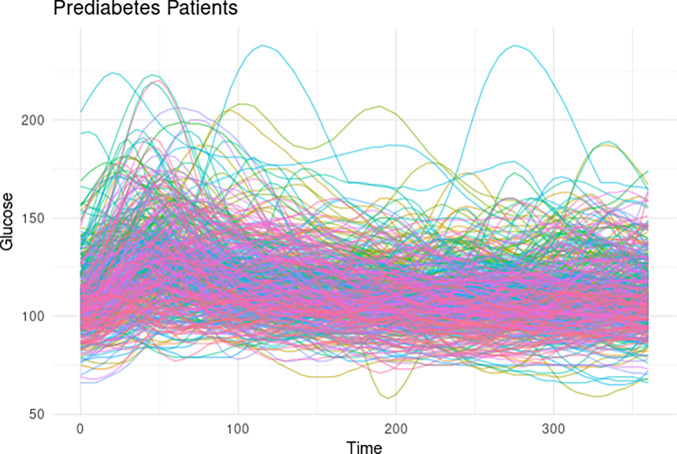
Glycemic responses six hours (360 minutes) post-dinner in all prediabetic individuals of AEGIS study, where color indicates participant.

Table 1 summarizes the scalar predictors used in our functional multilevel regression analysis. Distribution summaries are provided for all continuous variables to characterize the study population. Figure 2 illustrates all functional observations collected across different days from prediabetic patients, highlighting the significant heterogeneity of the data. This variability underscores the need for developing specific methods to address the extensive intra- and inter-individual differences evident in the repeated functional observations.

#### 2.3.1 Ethical Considerations

The study procedures adhered to ethical standards, with informed consent obtained from all participants. The study was approved by the Regional Ethics Committee (Comité Ético de Investigación Clínica de Galicia, registration code: 2012/025) and was conducted in accordance with the Helsinki Declaration.

## 3 Functional Models for Postprandial Glucose Responses

The AEGIS study provides an opportunity to examine glycemic response to diet in real-time while accounting for participant characteristics. In this paper, we restrict our attention solely to meals identified by participants as ”dinners” and to a time window of six hours after the meal. The clinical outcome is the functional multilevel process $Y_{ij}(t)$, t∈[0,360], for participant i∈{1,...,n} and day $j\;\in \;\{1,...,J_{i}\}$, where the number of days recorded $J_{i}$ is specific to the individual. As not all of the participants without diabetes reported dinners, n=377 and $J_{i}\in \;\{2,\;3,\;4,\;5,\;6\}$ in this application.

### 3.1 Objectives of the Functional Analysis methods in modeling the diet glucose response

The goal of our modeling strategy is to provide quantitative methods to answer common scientific questions that appear in clinical research when trying to understand the dynamics of postprandial glucose over time.

1. **Modes of data variability hetereogenity:** We consider a multilevel functional principal components model (unsupervised) to understanding the functional modes of variability in glucose trajectories across individuals and days.
2. **Functional response regression modeling:** We are interested in quantifying the statistical association between individual characteristics e.g. sex, age, diet and glucose response curves. For this purpose, we consider a function-on-scalar regression model (supervised).
3. **Predict Clinical Outcomes with Multilevel Postprandial Functional Information:** The postprandial glucose response provides a unique signature of individual glucose homeostasis Jagannathan et al. (2020). We propose a novel algorithm to incorporate information into a participant-level latent representation useful for the prediction of outcomes.
4. **Predictive Capacity Assessment:** Quantifying how well the model explains the observed data is critical for understanding model limitations and advantages. We propose a novel concept of R-squared for both supervised and unsupervised multilevel functional models.

### 3.2 Unsupervised Functional Models

We first introduce some notation. Denote by $Y_{ij}(t)$, t∈[0,360], the functional trajectory for individual i=1,…,n during period $j=1,\ldots ,J_{i}$ in the standart functional space $L^{2}([0,\;360])$. For notation simplicity, we assume that $J_{i}=J$ for all i=1,···,n, though methods can account for a different number of observations per study participant. Consider the following multilevel functional principal components analysis (MFPCA) model for $Y_{ij}(\cdot )$ from Di et al. (2009).


(1) $$Y_{ij}(t)=\mu (t)+\nu_{j}(t)+U_{i}(t)+W_{ij}(t)$$

In this model, μ(t) is the global mean, $\mu (t)+\nu_{j}(t)$ is the mean during time-period j, $U_{i}(t)$ is the subject-specific deviation from the visit-specific mean function, and $W_{ij}(t)$ is the residual subject- and period-specific deviation from the subject-specific mean. Here μ(t) and $\nu_{j}(t)$ are treated as fixed functions, and we assume that $U_{i}(\cdot )∼GP\left(0,\Sigma_{i}\right)$ and $W_{ij}(\cdot )∼GP\left(0,\Sigma_{ij}\right)$ are mutually uncorrelated zero mean Gaussian process with positive definite covariance operators $\Sigma_{i}$ and $\Sigma_{ij}$ respectively. These operators are defined in the functional space $L^{2}([0,\;360)]⊕L^{2}([0,\;360])$.

In the original work by Di et al. (2009), emphasis was placed on estimating the structures of the random processes $U_{i}(t)$ and $W_{ij}(t)$ through the Karhunen-Loeve decomposition. This method involves employing eigendecomposition to analyze within and between-group variability. For instance, for each individual i, the random function $U_{i}(t)$ can be expressed as $U_{i}(t)=\sum_{j=1}^{\infty }\;a_{ij}\phi_{j}(t)$, where ${\left\{\phi_{j}(\cdot )\right\}}_{j=1}^{\infty }$ are the eigenfunctions associated with the individual level, and ${\left\{a_{ij}\right\}}_{j=1}^{\infty }$ are the corresponding scores for the *i*^*th*^ individual.

In the original reference Di et al. (2009), a Bayesian modeling approach using Markov Chain Monte Carlo (MCMC) was proposed to estimate the scores. In a recent paper, Cui et.al, Cui et al. (2022), proposed a new scalable algorithm for estimating the eigenfunctions, eigenvalues, and scores that can be accessed through the <monospace>mfpca.face</monospace> function in the <monospace>refund</monospace> package by Goldsmith et al. (2020).

In this study, we focus on describing the diverse modes of glucose trajectory variability in terms of the eigenfunctions and eigenvalues, facilitating comprehensive understanding of the data structure.

### 3.3 Supervised function-on-scalar regression models

In addition to the functional postprandial glucose response $Y_{ij}(t)$, we observed covariates such as demographics, HbA1c, and meal-level dietary information. These predictors can be incorporated in the model’s fixed and/or random effects structure. To account for these covariates we consider models of the following type.


(2) $$Y_{ij}\left(t\right)=\sum_{l=1}^{L}X_{ij,l}\beta_{l}\left(t\right)+\sum_{k=1}^{K}Z_{ij,k}U_{i,k}\left(t\right)+W_{ij}\left(t\right),$$

Within this model, $X_{ij,l}$ are L fixed effects covariates, $\beta_{l}(t)$ is the fixed effect functional coefficient over t∈[0,360], $Z_{ij,k}$ are K random effects covariates, $U_{i,k}(t)$ is a random functional effect corresponding to subject i at time t, and $W_{ij}(t)$ is the residual variation that is unexplained by either the fixed or random effects. We assume that the $U_{i,k}(\cdot )$ and $W_{ij}(\cdot )$ processes are zero mean square integrable processes, with $W_{ij}(\cdot )$ being uncorrelated with all $U_{i,k}(t)$, though $U_{i,k}(t)$ can be correlated among themselves.

Models such as (2) have been proposed in the literature before and are easy to write down, but they are difficult to fit in larger data applications. To address this problem, we adapt the recently proposed fast univariate inference (FUI) for longitudinal functional data analysis proposed by Cui et al. (2021). This approach can be implemented by fitting many pointwise mixed effects models and then smoothing the fixed effects parameters over the functional domain. An important advantage of the approach is that it generalizes the intuition of fitting mixed effects models at every point over the domain of the temporal glucose trajectories. The core steps of the algorithm are indicated bellow:

1. For each point $t\in T_{m}$, fit a separate point-wise linear mixed model using standard multilevel software, that is
$$Y_{ij}\left(t\right)=\sum_{l=1}^{L}X_{ij,l}\beta_{l}\left(t\right)+\sum_{k=1}^{K}Z_{ij,k}U_{i,k}\left(t\right)+W_{ij}\left(t\right).$$
2. Smooth the estimated fixed-effects coefficients ${\tilde{\beta }}_{l}(t)$ using a linear smoother ${\hat{\beta }}_{l}(t)=S_{l}{\tilde{\beta }}_{l}$, where $S_{l}$ is a smoother that may or may not depend on l.
3. Use a bootstrap of study participants to conduct model inference:
  - (a) Bootstrap the study participants *B* times with replacement. Calculate ${\hat{\beta }}_{l}^{b}(t)$, the estimator of $\beta_{l}(t)$ conditional on the b=1,...,B bootstrap sample.
  - Arrange the ${\hat{\beta }}_{l}^{b}(t)$ estimators in a B×P (bootstrap samples by probabilities) and obtain the column mean ${\overset{‾}{\beta }}_{l}(t)$ and variance $v_{l}(t)=Var\left\{\beta_{l}(t)\right\}$ estimators.
  - Conduct a Functional Principal Component Analysis (FPCA) on the B×P dimensional matrix, extract the top Q eigenvalues $\lambda_{1l},\ldots \lambda_{Ql}$ and corresponding eigenvectors $\gamma_{1l},\ldots \gamma_{Ql}$.
  - For $n\;=\;1,...,N_{s}$ do
    - Simulate independently $\xi_{nq}∼𝒩\left(0,\lambda_{ql}\right)$ for q=1,…,Q. Calculate ${\hat{\beta }}_{l,n}(p)={\overset{‾}{\beta }}_{l}(p)+\sum_{q=1}^{Q}\;\xi_{nq}\gamma_{ql}$.
    - Calculate $u_{nl}={max}_{t\in [0,360]}\;\left\{\left|{\hat{\beta }}_{l,n}(t)-{\overset{‾}{\beta }}_{l}(t)\right|\sqrt{v_{l}(t)}\right\}$
  - Obtain $q_{1-\alpha ,l}$ the (1−α) empirical quantile of the $\left\{u_{1l},\ldots ,u_{N_{s}l}\right\}$ sample.
  - The joint confidence interval at *t* is calculated as ${\hat{\beta }}_{l}(t)\pm q_{1-\alpha ,l}\sqrt{v_{l}(t)}$.

### 3.4 Predicting Clinical Outcomes using Latent Representations

After fitting the models discussed in Section 3.3, residuals were used to define latent clusters of patients according to outlier behaviors. First, we outline the method employed to compute the residuals.

Consider a patient i=1,...,n, with their corresponding repeated observations j=1,...,J. The functional residual for each patient and observation was defined as:

(3) $${\hat{\epsilon }}_{ij}(t)=Y_{ij}(t)-{\hat{Y}}_{ij}(t),$$

where $Y_{ij}(t)$ denotes the observed glucose levels at postprandial time t∈[0,360], and ${\hat{Y}}_{ij}(t)$ is the estimated glucose level using only the fixed effects in the multilevel regression model:

(4) $${\hat{Y}}_{ij}\left(t\right)=\sum_{l=1}^{L}X_{ij,l}{\hat{\beta }}_{l}\left(t\right).$$

We endeavored to categorize the functional trajectories into three distinct cases based on these residuals: (i) **Stable Residuals**—${\hat{\epsilon }}_{ij}\approx 0$, for all t∈[0,360], indicating that the patient’s behavior is close to the conditional mean value; (ii) **Positive Deviations**—functional residuals with significant positive deviations suggest that the glucose values are larger than predicted, signifying potentially inadequate glucose management; (iii) **Negative Deviations**—trajectories with negative deviations leading to glucose levels below the expected range early in the postprandial window. Generally, this could be viewed as beneficial, indicating better glucose control, but it could also result in episodes of hypoglycemia.

As ${\hat{\epsilon }}_{ij}(t)=Y_{ij}(t)-{\hat{Y}}_{ij}(t)$ are random functions in $L^{2}([0,\;360])$, we apply multilevel functional PCA (as discussed in Section 3.2) to form a vector representation of the functional residuals, facilitating clinical outcome prediction.

For ${\hat{\epsilon }}_{ij}(t)$, we consider a model with components akin to (1):

(5) $${\hat{\epsilon }}_{ij}(t)=\mu (t)+\nu_{j}(t)+U_{i}(t)+W_{ij}(t),$$

where $U_{i}(t)=\sum_{j=1}^{\infty }\;a_{ij}\phi_{j}(t)$, with ${\left\{\phi_{j}(\cdot )\right\}}_{j=1}^{\infty }$ representing the random participant-level eigenfunctions, and ${\left\{a_{ij}\right\}}_{j=1}^{\infty }$ being the associated scores for participant i.

In practice, it’s crucial to estimate and truncate these coordinates to a finite number, ${\hat{a}}_{i}=\left({\hat{a}}_{i1},\ldots ,{\hat{a}}_{im}\right)$ in $R^{m}$. These coordinates succinctly encapsulate the functional information for each individual, and the associated eigenfunctions indicate the types of features present in the residuals (stable, positive, negative). Given a scalar outcome Z∈R (e.g., the HOMA-IR surrogate marker for insulin resistance) and other fixed-effect patient characteristics $X=\left(X_{1},\ldots ,X_{p}\right)\in R^{p}$, we consider a regression model:

(6) $$Z=g\left(\hat{a},\;X\right)+\epsilon ,$$

where ϵ is the random error with E(ϵ)=0, and g(⋅) is the conditional mean function. In our case, we assume $g(\hat{a},X)=\sum_{i=1}^{m}\;\gamma_{i}{\hat{a}}_{i}+\sum_{j=1}^{p}\;\beta_{j}X_{j}$ for coefficients $\gamma_{i}$, $\beta_{j}$. Other functional forms, such as additive or non-parametric models, could also be adopted.

### 3.5 Conditional and Unconditional $R^{2}$ in Multilevel Functional Models

The r-square coefficient, denoted as $R^{2}$, is the classical metric in statistical literature used to quantify the proportion of variance explained in a response variable by a set of corresponding predictors. Here, we focus on extending the $R^{2}$ coefficient for mixed functional models, considering pointwise marginal and conditional versions for supervised models as well as both pointwise and global versions for unsupervised MFPCA.

For each t∈[0,360] and i=1,...,n,j=1,...,J, we denote ${\tilde{Y}}_{ij}(t)$ and $Y_{ij}(t)$ as the predicted and observed functional trajectories, respectively. At any given time point t∈[0,360], the pointwise ${\tilde{R}}^{2}(t)$ for supervised models can be estimated using the standard univariate approach as follows:

(7) $${\tilde{R}}^{2}\left(t\right)=1-\frac{\sum_{i=1}^{n}\sum_{j=1}^{J}{\left(Y_{ij}\left(t\right)\;-\;{\tilde{Y}}_{ij}\left(t\right)\right)}^{2}}{\sum_{i=1}^{n}\sum_{j=1}^{J}{\left(Y_{ij}\left(t\right)\;-\;\bar{Y}\left(t\right)\right)}^{2}},\;\text{where}\;\bar{Y}\left(t\right)=\frac{1}{nJ}\sum_{i=1}^{n}\sum_{j=1}^{J}Y_{ij}\left(t\right).$$

We distinguish two scenarios in our modeling framework: supervised and subject-level unsupervised ${\tilde{R}}^{2}$. In the supervised framework, we define the marginal ${\tilde{R}}^{2}$, which includes only fixed effects, and the conditional ${\tilde{R}}^{2}$, which also incorporates random effects. The respective fitted values used to estimate $R^{2}$ are:

(8) $${\tilde{Y}}_{ij}^{marginal}\left(t\right)=\sum_{l=1}^{L}X_{ij,l}{\hat{\beta }}_{l}\left(t\right)$$

(9) $${\tilde{Y}}_{ij}^{conditional}\left(t\right)\sum_{l=1}^{L}X_{ij,l}{\hat{\beta }}_{l}(t)+\sum_{k=1}^{K}Z_{ij,k}{\hat{U}}_{i,k}(t).$$

In multilevel regression models, direct estimation of individual random effects is not always possible, especially in frequentist modeling. Nevertheless, leveraging their mean-zero property, as per structural assumptions, allows for the orthogonal decomposition of the mean square estimator. This approach facilitates the application of the standard $R^{2}$ formula, utilizing overall variance estimators for each random component. Generally, this is the method considered in standard univariate mixed-effect software.

For Bayesian multilevel models, the estimation of $R^{2}$ is direct and straightforward, as it circumvents the need to adjust for non-Gaussian random effects, a requirement in frequentist methods heavily reliant on traditional computational libraries for mixed effect modeling.

For individual unsupervised ${\tilde{R}}^{2}$, for an arbitrary patient ith and based on the Karhunen-Loève expansion, we define:

(10) $${\tilde{R}}_{i,k}^{2}\left(t\right)=1\;-\;\frac{\sum_{j=1}^{J}\;\;{\left(Y_{ij}(t)\;-\;{\tilde{Y}}_{ij}^{k}(t)\right)}^{2}}{\sum_{j=1}^{J}\;\;{\left(Y_{ij}(t)\;-\;{\overset{‾}{Y}}_{ij}(t)\right)}^{2}}$$

Where ${\tilde{Y}}_{ij}^{k}(t)=\hat{\mu }(t)+{\hat{\nu }}_{i}(t)+\sum_{j=1}^{k}\;{\hat{a}}_{ij}{\hat{c}}_{i}(t)$. The model estimation strategy provides direct access to individual random effects, essential for our general fitting framework (refer to Cui et al. (2022) for technical details).

The global estimator of ${\tilde{R}}^{2}$ for both supervised and unsupervised models is defined as the average of pointwise estimates over the time interval [0,360]:

(11) $${\tilde{R}}^{2}=\frac{1}{360}\int_{0}^{360}\;{\tilde{R}}^{2}\left(t\right),dt.$$

## 4 Application of Multilevel Functional Models to AEGIS

### 4.1 Unsupervised analyses

**Figure 3:**
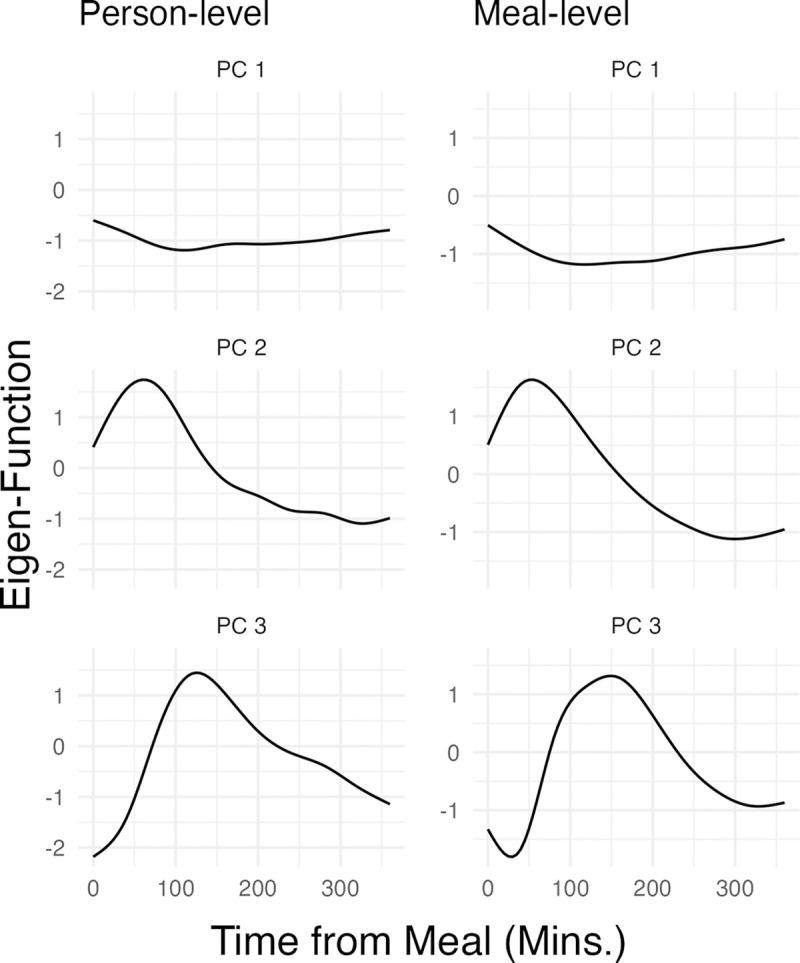
Eigen-Functions of the Meal Data at Both Levels

Following the methodology outlined in Section 3.2, we decomposed the postprandial glucose response functions for AEGIS individuals without diabetes into principal components. Figure 3 displays the primary eigenfunctions derived from the spectral decomposition of the random functions $U_{i}(\cdot )$ and $W_{ij}(\cdot )$. These functions represent the first and second levels of the hierarchical structure, respectively. Given the symmetry of the associated scores about zero, the eigenfunctions could equivalently be negated.

The first two eigenfunctions at the individual and meal levels in Figure 3 accounted for a substantial portion of the total variability — more than 80%. The first eigenfunctions at both levels suggested an almost time-invariant absolute level, with a relatively shallow concavity peaking at around 100 minutes. The second eigenfunctions contained a more pronounced peak at around 60–80 minutes after the meal. These were very similar across levels, not unexpected given mutual orthogonality not being enforced between levels.

**Figure 4:**
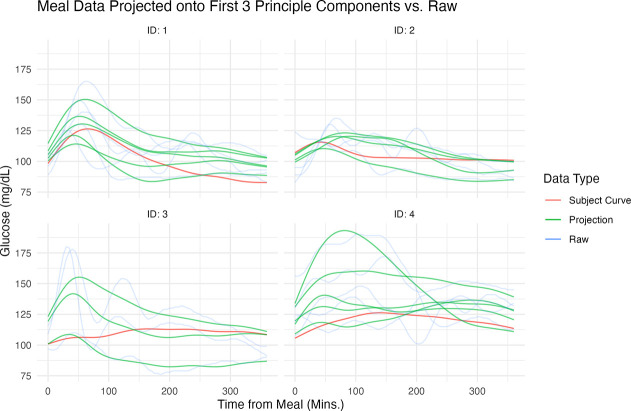
Projections of Raw Data onto Eigen-Functions. **Raw Trajectories (Transparent Blue):** These trajectories display the original data collected over consecutive days. **Estimated Participant Trajectories (Red Curve):** Utilizing the scores and eigenfunctions at the individual level, these visualizations illustrate the estimated participant-level trajectories. **Smoothed Projected Trajectory for Each Meal (Green Curves):** Constructed from the scores and eigenfunctions at both levels, these curves represent the sprojected meals from each participant.

Figure 4 shows the CGM raw trajectories for four randomly selected individuals – two with prediabetes and two who are normoglycemic – along with their projections onto the first and second hierarchical levels of the fit MFPCA model. In the projected space of the first three components, we observe pronounced data heterogeneity, particularly among the participants with prediabetes. This variability underscores the necessity of employing multilevel models to properly account for the data structure.

### 4.2 Supervised analyses

We first applied the function-on-scalar regression model introduced in section 3.3 with the covariates described in Table 1, a fixed intercept, and individual-level random intercepts, with the goal of examining time-dependent association between these covariates and the glucose response $Y_{ij}(t)$, t∈[0,360]. We included initial glucose concentration, measured 5 minutes prior to the recorded meal, to introduce some information related to prior conditions.

**Figure 5:**
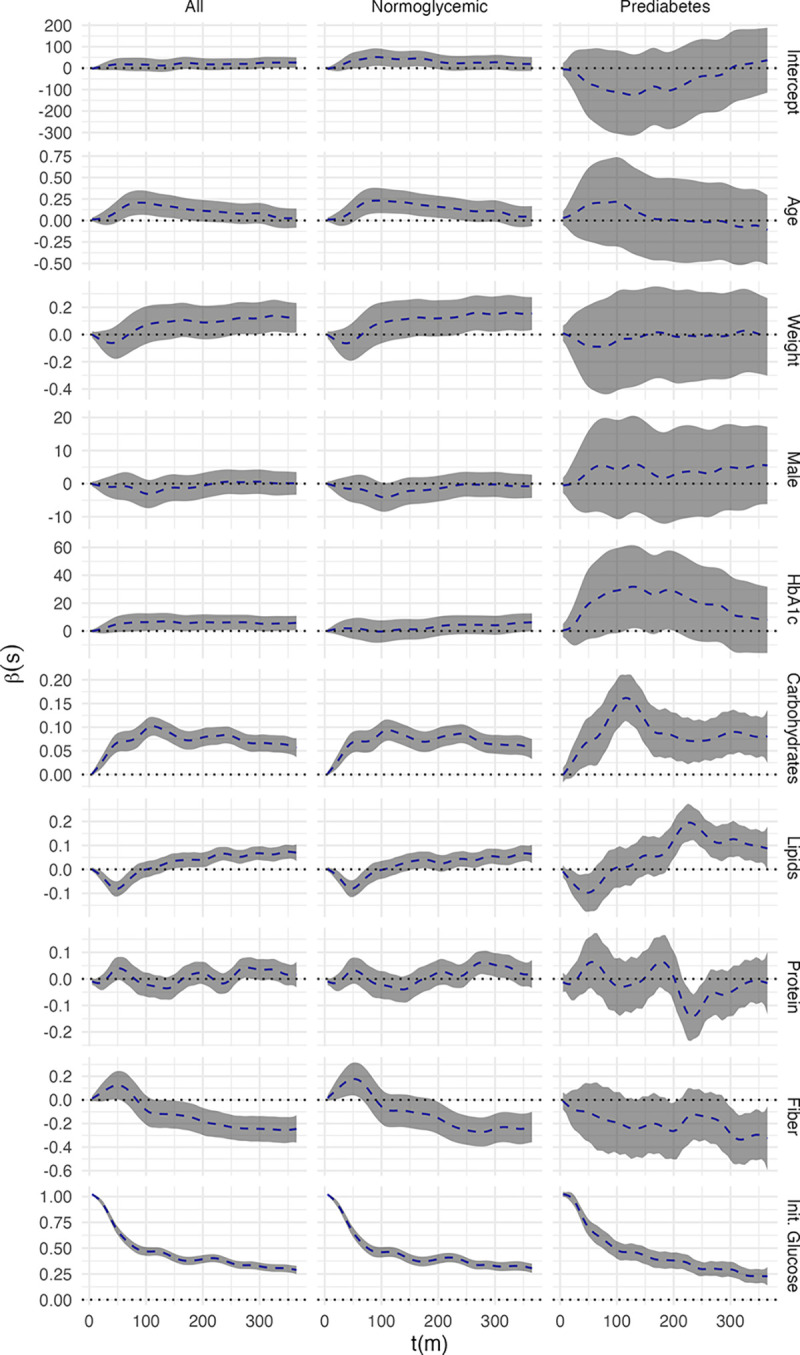
Relevant Covariate Function Estimates from FUI

Figure 5 displays the fixed effect coefficient functions with associated joint confidence intervals. Note that only those AEGIS participants which recorded dinners were included in these analyses. The first column indicated the coefficient functions for the entire AEGIS population which did not have diabetes (n=377), the second included just those labelled as normoglycemic (n=319), and the final column contained those individuals with prediabetes (n=58). Each plot was augmented with a dotted line at zero to make it easier to discern where point-wise and joint statistical significance are achieved. As can be seen in Figure 5, most covariates achieved pointwise significance over some interval in at least one population, but each had a unique coefficient function shape and subsequent interpretation.

Examining Figure 5, it was first apparent that estimates within the pre-diabetes populatio were more variable. This was logical given this subset’s smaller size and greater heterogeneity in glycemic regulation. With increasing age, there was an increase in postprandial glucose concentrations peaking at 90 minutes. This effect gradually declined until disappearing 5–6 hours after ingestion. No significant differences were observed between men and women. Heightened levels of A1c were associated with increase in glucose concentrations along the continuoum from normoglycemic to prediabetes, but not within groups. The greatest increase in postprandial glycemic response was observed in meals with higher amounts of carbohydrates, with greater effect in participants with prediabetes. An opposite effect was observed in meals with higher amounts of lipids, where initially (up to 50 minutes post-meal) there is a decrease in glucose concentrations, followed by a much later and more mild increase in glucose concentration. Again, this effect was greater in individuals with prediabetes than in normoglycemic individuals. Protein intake did not appear to have a notable effect on glucose concentrations. The intake of higher amounts of fiber seemed to have a buffering effect, significantly decreasing glucose concentration starting 3 hours after the meal. Initial blood glucose concentration had the greatest coefficient function magnitude, being particularly influential in the time directly after the meal. While this could be due to high temporal auto-correlation in the CGM data, the observed effect did not decay to zero over the course of the meal window. The higher initial glucose concentrations thus seemed to indicate postprandial glucose concentration in more than an autoregressive capacity.

### 4.3 Prediction of Clinical Outcome with Latent Residual Representations

We used the estimated residuals from a multilevel regression model to predict HOMA-IR after the baseline study period. HOMA-IR is a key indicator in human metabolism, closely linked to insulin resistance and the progresion from prediabetes to clinical diabetes mellitus in individuals with obesity.

Table 2 demonstrates the substantial improvement in predictive accuracy achieved by integrating the first two scores into a linear regression model for this continuous biomarker. The increase in predictive capacity is more noticable for those with prediabetes, likely due to the biological relevance of the HOMA-IR biomarker to diabetes progression. The base model here included baseline HbA1C, lab blood glucose, age, and sex.

### 4.4 Marginal and Conditional $R^{2}$ Analysis

**Figure 6:**
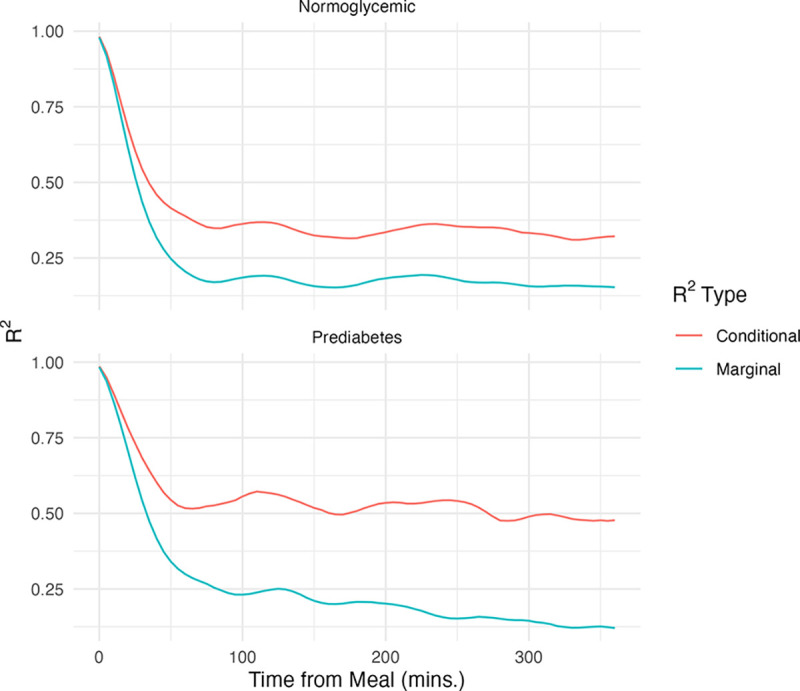
Marginal and Conditional R2 vs. PCA

We next assessed the explanatory capacity of the multilvel functional model with the new notion of mixed functional $R^{2}$, providing an estimate of the variance explained by the model over the entire relevant functional domain. Figure 6 was constructed to demonstrate both marginal and conditional $R^{2}$ functions for the function-on-scalar model. The unconditional $R^{2}$, representing variance explained by fixed effects, was plotted in red. The conditional $R^{2}$ on the other hand, including variability attributable to both fixed and random effects, was depicted using blue.

For normoglycemic participants, conditional and marginal $R^{2}$ values aligned closely in the first 50 minutes, indicating minimal influence of individual random effects post-adjustment for baseline glucose levels. Beyond this period, $R^{2}$ values stabilized with random effects contributing to a more than 50% increase in variability explained.

In prediabetic individuals, conditional and marginal $R^{2}$ diverged earlier in the postprandial period, potentially a result of the increased glycemic heterogeneity (or smaller sample size) of the participants with prediabetes.

Even including participant-specific random effects, the proportion of functional variability explained by our models remained limited, indicating the existence of more complex structure not captured by the covariates we have collected. In order to explain a greater degree of variability in the data by the model, incorporating additional variables or more complex random component structure maybe be necessary.

### 4.5 Summary of Results

Our models illustrated the impacts of different dietary and participant characteristics on postprandial glucose response curves over the entire temporal domain, including the importance of individual-specific random effect structures. These findings could facilitate the creation of personalized nutritional recommendations based upon the difference between an individual’s usual postprandial response and the ideal one. This could potentially be extended to populations with type 2 diabetes mellitus, given that the associations observed for individuals with prediabetes appear to be more extreme analogs to those for normoglycemic participants. The core scientific findings discussed in this section are outlined in Table 3.

## 5 Discussion

This paper introduces a functional data analysis framework for studying postprandial CGM response curves. Applying this framework to the AEGIS study yielded novel insights, particularly the differential glycemic response to increased lipid intake between normoglycemic participants and those with prediabetes.

An important strength of our modeling framework is that the methods are computationally scalable and can be applied in large medical cohort studies, such as those currently ongoing in Israel and the USA Shilo et al. (2021), The “All of Us” Research Program (2019). Another strength is that we analyze a random sample of the general population, unlike the mentioned studies, which are observational in nature and involve specific participants with risk of selection bias.

The methods discussed here could also be used to model oral glucose tolerance test data Jagannathan et al. (2020). However, our use of CGM data addresses the scientific question with a greater deal of generality, as CGM monitor patients in more realistic, free-living conditions.

There is a large body of literature modeling glycemic responses to food intake. Many existing models are based on large systems of differential equations or time series models where the functional form of the model is specified with expert biological knowledge Bergman (2021), Urbina et al. (2020), Maas et al. (2015), Shi et al. (2020), De Gaetano et al. (2021), Eichenlaub et al. (2021), Zhang et al. (2016), Eichenlaub et al. (2019), Trajanoski & Wach (1996), Holtschlag et al. (1998). In contrast, our approach is fully data-driven with time-dependent semi-parametric associations. A potential advantage of our semi-parameteric models is that we can interpret the impact of meal intake with a β– functional coefficient, and have robust estimation and inference within the multilevel data structure. For future work, we propose a new extension of the glucotype concept Hall et al. (2018b) based on multilevel functional models, modeling the conditional variability response rather than the conditional mean response.

## Figures and Tables

**Table 1:** Description of the variables used in the AEGIS. Distribution summaries for continuous variates include first Mean (Standard Deviation), followed by the Median [Min., Max.].

Variate	Description	Distribution Summaries	
		Normoglycemic (N=319)	Prediabetes (N=58)
**Individual Level**			
Age (yrs)	Participant age at screening	44.6 (13.7) 44.0 [18.0, 81.0]	58.7 (12.0) 61.0 [23.0, 84.0]
Weight (kg)	Participant weight at screening	73.7 (14.3) 72.5 [41.0, 130]	83.0 (19.6) 79.2 [49.0, 145]
Gender	Participant reported gender	Male: 121 (37.9%) Female: 198 (62.1%)	Male: 18 (31.0%) Female: 40 (69.0%)
HbA1c (%)	Baseline glycosylated hemoglobin	5.25 (0.25) 5.30 [3.10, 5.60]	5.86 (0.20) 5.80 [5.70, 6.40]
**Meal Level**			
Carbohydrates (g)	Self-reported and dietition reconstructed meal carbohydrates	59.9 (40.5) 52.3 [0, 513]	53.7 (37.5) 45.7 [0, 226]
Lipids (g)	Self-reported and dietition reconstructed meal fats	30.1 (23.8) 25.4 [0, 237]	25.7 (22.3) 21.8 [0, 169]
Proteins (g)	Self-reported and dietition reconstructed meal protein	27.5 (17.9) 24.2 [0, 200]	25.9 (17.0) 23.1 [0.4, 105]
Fiber (g)	Self-reported and dietition reconstructed meal fiber	8.8 (6.7) 7.2 [0, 89.3]	9.1 (7.0) 8.1 [0, 63.1]
Initial Glucose (mg/dL)	CGM glucose at beginning of meal	103 (15.3) 101 [52, 237]	110 (19.4) 107 [56, 196]

**Table 2:** Associations and added predictive capacity for residual scores with follow-up HOMA-IR.

Model Feature	Population	
	Normoglycemic	Pre-Diabetes
Score 1 Coef. (SE)	−0.386 (0.234)	−1.03 (0.431)
Score 2 Coef. (SE)	−0.500 (0.244)	−0.128 (0.441)
Base Model *R*^2^	5.39 × 10^−3^	0.072
Model including Scores *R*^2^	4.23 × 10^−2^	0.269

**Table 3:** Summary of Findings

Result	Implication
There is substantial heterogeneity in level and shape of postprandial glucose curves both between and within individuals	Appropriately accounting for the hierarchical structure of the postprandial responses is required for adequate explanation of the observed glucose patterns
The functional beta coefficients for different macro and micro-nutrients are not time-invariant, and they vary in intensity and direction.	Postprandial glucose response is influenced by the composition of macro and micro-nutrients in distinct ways, with interactions between dietary components.
The functional beta coefficients diet components differ between normoglycemic and prediabetic individuals.	Metabolic responses to the same diet differ between normoglycemic and prediabetic patients, indicating the importance of glycemic capacity in formulating diet recommendations.
The participant-level eigenfunction scores contribute substantially to the variability explained in HOMA-IR over a model including just demographic features, though the final R-square is still somewhat low.	Embedding postprandial glucose responses shows promise as a means of forming latent subgroups which are predictive of metabolism-related outcomes, but a further modelling may be required.
The *R*^2^ functional mixed model explainability metrics are not time-invariant, showing a decline over time, and random effects significantly increase the variability explained in the predictions 50 min after post-meal intake.	Post-meal functional response analysis indicates significant individual heterogeneity, necessitating alternative, perhaps more personalized, model structures.
