# Supplementary material for: Beyond Scalar Metrics: Functional Data Analysis of Postprandial Continuous Glucose Monitoring in the AEGIS Study
Source: ArXiv. 2025 Sep 1:arXiv:2405.14690v2. Originally published 2024 May 23. Preprint. [Version 2] (PMC11142320)
Supplement: Supplement 1 [file NIHPP2405.14690v2-supplement-1.pdf]

Appendix A AEGIS R^2

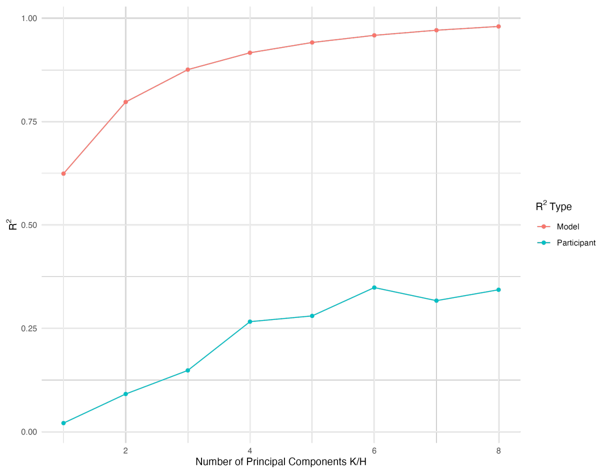

Fig. A1: Participant-level and model-level global R^2 estimates for MFPCA models by number of eigenfunctions K, H at both levels

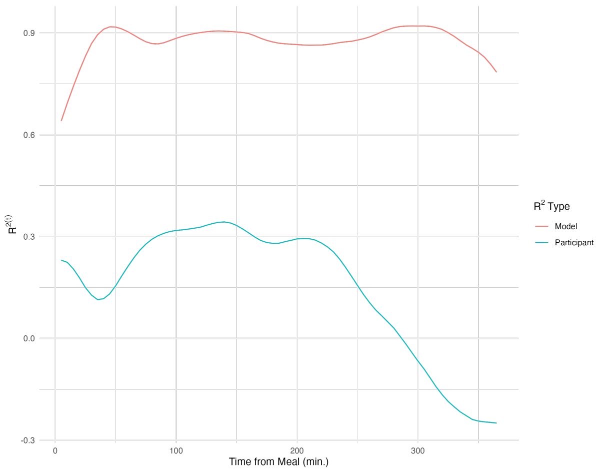

Fig. A2: Participant-level and model-level $\widetilde{R}^2$ estimates for the chosen MFPCA model with $K = H = 3$ eigenfunctions at both levels

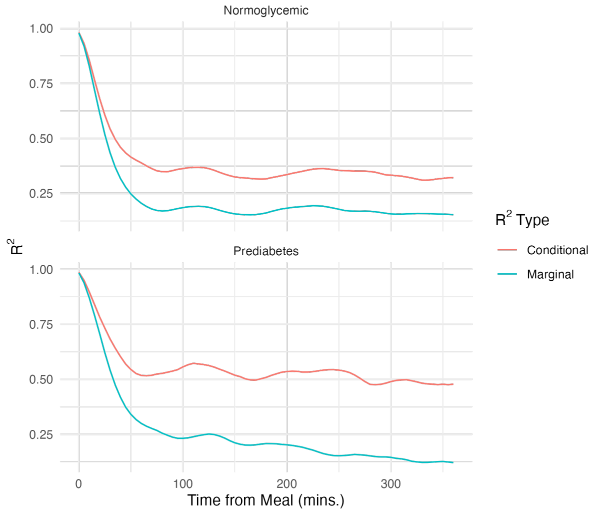

Fig. A3: Marginal and Conditional R^2 plotted over the course of the postprandial observation window
